# Timely evidence generation in epidemic response: lessons from an attempted analytical epidemiological investigation during an mpox outbreak, Ireland, 2022

**DOI:** 10.2807/1560-7917.ES.2026.31.39.2600170

**Published:** 2026-10-01

**Authors:** Charlotte Salgaard Nielsen, Eve Robinson, Patricia Garvey, Kate O’Donnell, Noel McCarthy, Randal Parlour, Fiona Lyons, Derval Igoe

**Affiliations:** 1 Health Service Executive (HSE)-Health Protection Surveillance Centre (HPSC), Dublin, Ireland; 2 ECDC Fellowship Programme, Field Epidemiology path (EPIET), European Centre for Disease Prevention and Control (ECDC), Stockholm, Sweden; 3 Institute of Population Health, Trinity College Dublin, Dublin, Ireland; 4 Health Service Executive (HSE) Public Health: National Health Protection Office, Dublin, Ireland; 5 Health Service Executive (HSE) Sexual Health Programme, HSE Health & Wellbeing, Strategy and Research, Healthcare Strategy, Dublin, Ireland

**Keywords:** Disease Outbreaks, Emergency Preparedness, Governance, Mpox (monkeypox), Stakeholder Engagement, Surge Capacity

## Abstract

The emergence of mpox cases in Ireland in May 2022 triggered an urgent public health response, including an attempted analytical epidemiological investigation aimed at informing vaccination strategies and risk communication, particularly among gay, bisexual and other men who have sex with men. At the time of implementation, case numbers had declined to an extent that limited recruitment, resulting in early suspension of data collection. We present a structured reflection of the investigation, drawing on a lessons learnt review and the experiences of those involved, to offer actionable recommendations to strengthen evidence generation in future outbreak preparedness and response efforts. Key lessons include the critical importance of stakeholder engagement, clear governance and legal frameworks, and the need for enhanced procedural preparedness and pre-established surge capacity arrangements to enable rapid analytical investigations during health emergencies. These insights align closely with Ireland’s National Health Protection Strategy and the extended mandate of the European Centre for Disease Prevention and Control. Beyond existing international frameworks for outbreak preparedness, this reflection highlights the practical implementation barriers that must be addressed to enable timely and coordinated epidemiological investigations across Europe.

## Background

Ireland’s emergency response framework operates through a multi-tiered governance system involving institutions such as the Department of Health and the Health Service Executive (HSE), the latter of which includes the National Health Protection Office (NHPO) and the Health Protection Surveillance Centre (HPSC). The HSE, Ireland’s publicly funded healthcare system, serves as the operational lead for health service delivery and emergency response. The HPSC plays an essential role in national infectious disease surveillance and outbreak investigation, serving as a central hub for epidemiological intelligence and technical expertise during health emergencies.

In May 2022, the emergence of mpox cases in countries without previously recognised endemic transmission, including Ireland [1,2], highlighted gaps in national preparedness, including the capacity to rapidly generate epidemiological evidence to guide response efforts. In response to the scale of the outbreak and the subsequent declaration of a Public Health Emergency of International Concern (PHEIC), the HSE established a national crisis management team to coordinate clinical, public health, vaccination, communication and research efforts. Facing limited vaccine supply, the HPSC led the initiation of an analytical epidemiological investigation to generate early evidence and inform Ireland’s mpox vaccination strategy. The HPSC and partnering organisations encountered substantial implementation barriers, ultimately contributing to the early suspension of the investigation. This manuscript presents a structured reflection on the efforts to establish and initiate the investigation. The identified lessons were initially derived through a retrospective review, informed by project documentation, investigation timelines, and correspondence supplemented by discussions and reflections among those involved in the planning and initiation of the investigation. They were documented in an internal report and later refined in the context of outbreak preparedness. 

Similar challenges in generating epidemiological evidence during public health emergencies have been described internationally. Guidance from the World Health Organization (WHO) and the European Centre for Disease Prevention and Control (ECDC) emphasises the importance of preparedness planning, standardised investigation protocols and rapid mobilisation mechanisms [3,4]. However, less attention has been given to the operational realities affecting successful implementation during evolving outbreaks. 

This article provides practical insights into the barriers that we encountered when attempting to establish an analytical epidemiological investigation during an evolving public health emergency, and identifies lessons for strengthening preparedness and the capacity to generate timely evidence during future emergencies.

## Investigation timeline and key milestones

To contextualise, we present a timeline of mpox cases reported through the national Computerised Infectious Disease Reporting (CIDR) system in Ireland, highlighting key milestones related to the analytical epidemiological investigation (Figure). The first mpox case in Ireland was notified on 31 May 2022 [2], and the analytical investigation was first proposed on 18 July 2022 (Figure). Existing partnerships were leveraged to convene an advisory group to collaboratively design a prospective multi-site case–control study, planned to be conducted in three sites providing infectious disease and sexual health services in Dublin. The first meeting of the advisory group took place in the last week of July 2022, followed by extended discussions and preparatory activities. These included establishing governance arrangements across participating organisations, clarifying the legal basis for the investigation, obtaining site agreements, finalising operational procedures, and addressing logistical requirements related to recruitment, survey implementation and data collection. In the absence of pre-established pathways for rapid activation of an analytical investigation, these processes required substantial coordination and contributed to an interval of almost 4 months from proposal of the investigation in July 2022 to commencement of data collection in mid-November 2022. By this time, case numbers had declined substantially, and the investigation was suspended 1 month later with only one case and five controls enrolled. Reaching the target sample size of 45 cases and 135 controls was no longer considered feasible. The advisory group discussed reinitiating data collection in the event of a later surge in cases; however, this did not become relevant, as case numbers remained persistently low within a timeframe suitable for timely evidence generation. 

**Figure:**
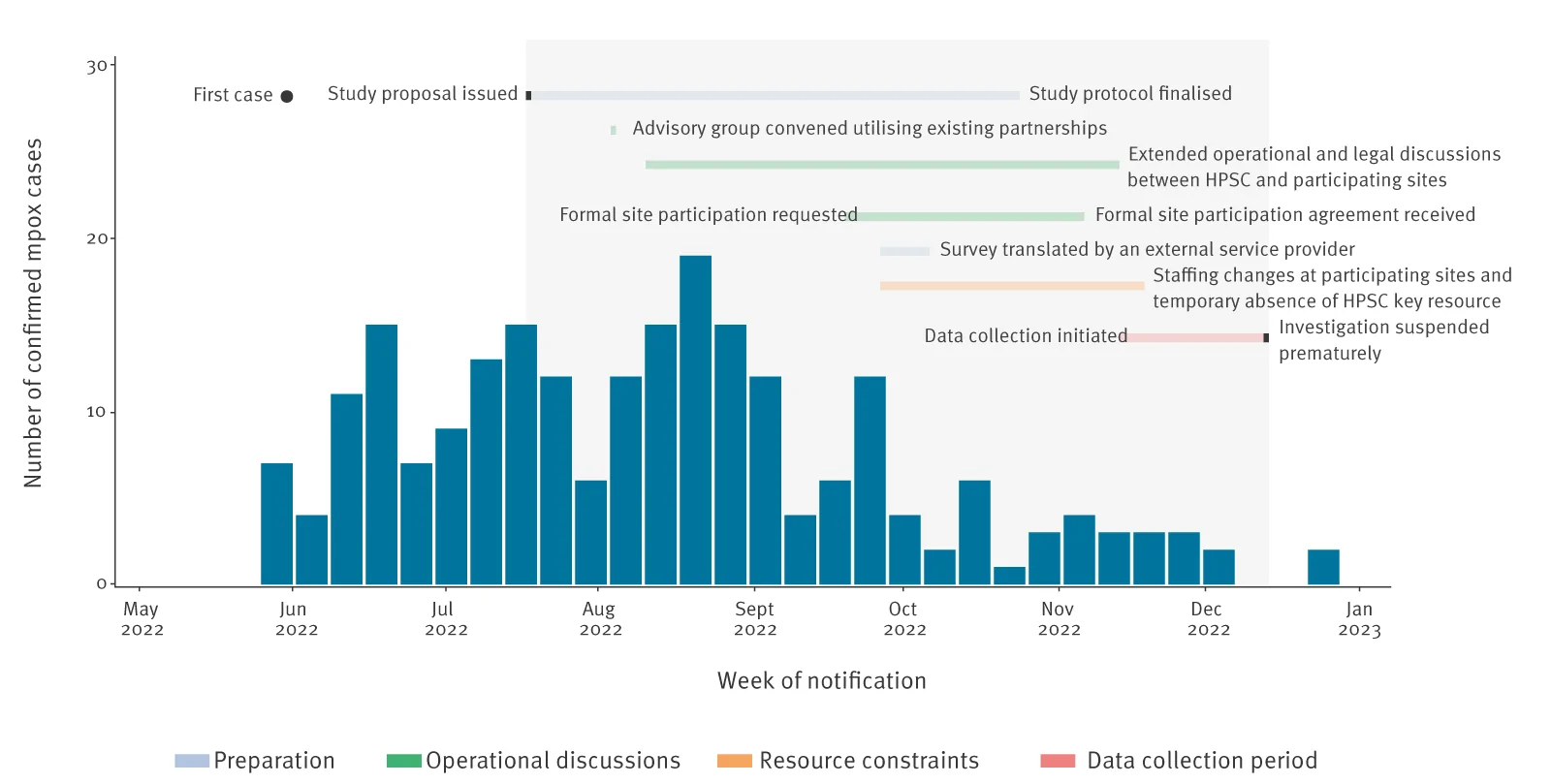
Weekly number of notified confirmed mpox cases following emergence, with highlighted key events during an attempted analytical epidemiological investigation, Ireland, May–December 2022 (n = 100)

HPSC: Health Protection Surveillance Centre.

Related activities are colour-coded.

## Lessons identified

Drawing on the investigation milestones presented above (Figure), this section details the key lessons identified. Practical preparedness and implementation checklists for each thematic area are provided in the Supplement.

### Collaboration and stakeholder engagement

**What we found:** A cornerstone of the planning and commencement of the investigation was the robust collaboration between HPSC, infectious disease and sexual health services, non-governmental organisations (NGOs) and academic institutions. Investment in long-term relationships with relevant community actors – developed through responses to previous outbreaks among gay, bisexual and other men who have sex with men (gbMSM) and through broader efforts to prevent and respond to sexually transmitted infections (STIs) – enabled culturally sensitive approaches and the use of community-appropriate language. While these partnerships are essential for effective community engagement and participation in public health investigations, limited human and operational resources, including staff time and capacity to coordinate stakeholder engagement, constrained the ability to operationalise stakeholder involvement and delayed the start of the investigation.

**Lessons identified:** Effective investigations require early, structured and sustained stakeholder engagement supported by adequate and continuous investment. While trusted partnerships may facilitate collaboration during emergencies, their existence alone does not ensure sufficient capacity for timely engagement. In our experience, competing priorities, limited personnel availability and time constraints can hinder the sustained coordination required for an effective investigation. Maintaining the capacity to engage across organisational boundaries therefore requires ongoing collaboration, clear communication and continued investment before and during emergencies.

### Governance and legal clarity

**What we found:** The investigation revealed gaps in awareness and operationalisation of Ireland’s governance framework for emergency investigations. The absence of pre-established protocols for such investigations within preparedness plans contributed to delays. Consequently, the investigation was initially classified by some parties as 'research' rather than a statutory investigation under the Medical Officer of Health’s (MOH’s) legal mandate, resulting in a delay of up to 48 days in obtaining the necessary approvals from participating sites. However, under Irish Health Acts 1947 and 1953, Infectious Disease Regulations 1981 and subsequent amendments [5], the MOH has statutory authority to investigate and control notifiable infectious diseases and outbreaks as part of routine public health practice. Data collected for these purposes are intended to support immediate public health action rather than to generate generalisable scientific knowledge. This distinction was not widely understood across all involved parties. The delay was further compounded by the sensitive nature of the investigation, including survey questions on sexual behaviours, which required additional liaison with participating sites to set out the legal and ethical basis for collecting these data under the MOH regulations.

**Lessons identified:** Ambiguity in legal and governance frameworks can delay investigations considerably. Clear communication of statutory authority and early engagement with governance structures are essential for timely action. We developed communication materials clarifying the legal mandate during the investigation and made these available as part of an HPSC toolkit. Further, a dedicated resource outlining the role and legal remit of the MOH statutory function, specifically relating to outbreak response, is now publicly available on the HPSC website [6].

### Operational readiness and investigation logistics

**What we found:** The investigation encountered several procedural and logistical challenges. Protracted discussions to secure formal agreements from participating sites slowed preparations for the recruitment of cases and controls and subsequent data collection. Technical challenges also emerged, including issues with survey and platform configuration, as well as the activation of links distributed via text message. While translation services were available, the need to translate the survey into languages other than English had not been identified as a potential bottleneck during the preparation phase. This task was outsourced to an external service provider and, given the complexity of the questions, further slowed the survey distribution.

**Lessons identified:** Operational bottlenecks – especially around translation and digital platforms – can critically slow investigations. These challenges highlight the need for pre-translated survey instruments and pre-tested tools, ready-to-deploy resources, and trained technical personnel as integral components of preparedness planning.

### Preparedness planning and anticipation of early evidence needs

**What we found:** In the years preceding the 2022 mpox outbreak, Ireland had not conducted simulation exercises specifically focused on rapidly undertaking analytical investigations in emergencies. Combined with insufficient workforce and operational capacity planning, this limited the procedural readiness of authorities to quickly reallocate resources and coordinate investigations effectively under emergency conditions. Reflecting on the early stages of the outbreak, it became clear how important it is to proactively identify and prioritise evidence needs early in the response.

**Lessons identified:** Preparedness planning that explicitly integrates rapid analytical investigation capacity – and anticipates early evidence needs – is essential for agile and effective outbreak response. Global initiatives such as the WHO Unity Studies [7] demonstrate the value of inter-pandemic preparedness through standardised investigation protocols and simulation exercises. Similar approaches have been incorporated into preparedness activities at European and national levels, including through national preparedness frameworks, dedicated rapid response capacities and outbreak investigation mechanisms designed to support timely evidence generation during emergencies [3,8]. However, the Irish experience demonstrates that availability of standardised protocols alone is insufficient; timely implementation also depends on operational factors including governance arrangements, legal clarity, stakeholder coordination and dedicated workforce capacity.

### Human resources and surge capacity planning

**What we found:** During the 2022 mpox outbreak, human capacity limitations were a substantial barrier to implementing analytical investigations. Staffing changes, temporary absences among key personnel and competing outbreak priorities left teams operating at or beyond capacity, with limited flexibility to carry out activities beyond their core response duties. Securing additional financial resources for recruiting additional staff was impractical from a time perspective during an international health threat. The co-occurrence of multiple emergencies in 2022 [1,9,10] particularly pressured health systems. Studies done after COVID-19 consistently indicate that workforce resilience, particularly during simultaneous health emergencies, is crucial for successful public health responses [11]. Although the teams involved were strongly motivated and dedicated to inform the mpox vaccination strategy, resource constraints within the HPSC, the NHPO and collaborating actors at various levels left little human capacity to implement the investigation. These challenges reflect broader system-wide difficulties in managing surge capacity amid concurrent public health threats.

**Lessons identified:** Effective outbreak investigations require dedicated human resources and surge capacity mechanisms that can be rapidly mobilised without compromising essential response activities. Workforce resilience and cross-sector collaboration are vital for maintaining capacity and flexibility during multiple or prolonged emergencies.

## European relevance and strategic implications

The challenges identified during this analytical investigation in Ireland reflect broader issues recognised in European and international preparedness frameworks. Guidance from the ECDC has emphasised the importance of timely outbreak investigations, strengthened surveillance capacities and coordinated response mechanisms, while WHO initiatives promote standardised approaches for rapid evidence generation during emerging health threats [3,7]. The lessons from the investigation align with strategic priorities outlined in Ireland’s Health Protection Strategy 2022–2027 [12] and the ECDC's extended mandate [13], particularly in the areas of collaboration, governance clarity and resilience as core preparedness pillars.

Recent reforms at European Union (EU) level, including the strengthened ECDC mandate and the establishment of the Health Emergency Preparedness and Response Authority (HERA) under the European Commission, reflect increased institutional attention to preparedness and response capacities. However, the experiences described here highlights that broader preparedness frameworks also need to be complemented by sufficient operational capacity to enable timely epidemiological investigations during public health emergencies.

While some findings are specific to Ireland, the broader operational challenges identified are likely to be relevant in other countries. This reflection complements existing preparedness frameworks by highlighting practical implementation barriers that may delay analytical epidemiological investigations even when relevant guidance is available. The key points for consideration arising from this reflection are summarised in the Box.

Box **Points for consideration to strengthen analytical epidemiological investigations during public health emergencies**

**Strengthen governance and legal clarity**

Develop and disseminate clear national guidance on statutory roles, approval pathways and legal frameworks for analytical investigations during outbreaks. Engage relevant governance structures and stakeholders early to expedite initiation and implementation.

**Enhance operational and technical preparedness**

Formalise investigation protocols within broader emergency response frameworks and maintain ready-to-deploy toolkits, including adaptable protocols, questionnaire modules, pre-translated materials, digital platforms and technical support to improve procedural clarity. Ensure expertise is embedded within teams to support rapid deployment and troubleshooting.

**Build human and surge capacity**

Establish multidisciplinary surge capacity mechanisms for rapid mobilisation, integrate analytical epidemiology into routine workplans, and establish priority-setting processes to rapidly reallocate staff during emergencies.

**Institutionalise preparedness**

Embed analytical investigations within preparedness planning through regular simulation exercises, standard operating procedures and routinely maintained investigation resources.

**Foster collaboration and continuous improvement**

Strengthen collaboration across public health authorities, healthcare services, academia and affected communities, while promoting knowledge sharing between countries and comparative analyses of outbreak preparedness and response mechanisms. Establish feedback loops to review investigation processes and incorporate lessons into future preparedness strategies.

## Discussion

The attempted analytical epidemiological investigation during the 2022 mpox outbreak in Ireland offers valuable insights into the structural and operational challenges that can hinder the initiation of analytical epidemiological investigations during an evolving public health emergency. These lessons underscore the necessity for building capacity to respond swiftly to emerging health threats. Earlier initiation during the growth and peak phases of the outbreak may have increased the likelihood of reaching the target sample size within a timeframe suitable for timely evidence generation. Strengthening legal and operational frameworks, investing in skilled personnel and digital infrastructure, and embedding formal investigative activities within preparedness and preparedness exercises are therefore important components towards improving readiness for future crises. 

This reflection has several limitations. Firstly, the lessons presented are derived from a single national experience during the 2022 mpox outbreak in Ireland and may not be directly transferable to all public health systems or outbreak contexts. However, many of the identified challenges relate to common elements of outbreak preparedness, including governance, operational readiness, stakeholder coordination and workforce capacity. Secondly, as this manuscript reflects on an investigation that was not completed, the lessons primarily relate to the processes and barriers affecting its initiation rather than the outcomes or findings of a completed analytical study. Thirdly, although lessons were informed by review of project documentation and reflections from individuals involved in the investigation, the process was not designed as a formal qualitative evaluation and may therefore not capture all perspectives or experiences. Finally, the rapidly evolving nature of the 2022 mpox outbreak and subsequent changes in preparedness frameworks mean that some recommendations may require adaptation as public health systems continue to evolve.

## Conclusion

By reflecting on the challenges encountered while attempting to establish an analytical epidemiological investigation during the 2022 mpox outbreak response in Ireland, this article highlights practical preparedness measures that can be addressed before future emergencies. These include clarifying governance, establishing mechanisms for rapid agreements and cross-organisational coordination, maintaining surge capacity and backup expertise, and ensuring that digital investigation tools and data collection processes can be rapidly deployed. Preparedness and simulation exercises could further test these operational components, including the ability to mobilise personnel, secure participation from relevant sites, and initiate investigations across organisational boundaries. Addressing these practical barriers in advance may improve the ability of public health systems to generate timely epidemiological evidence to inform response measures during future emergencies and strengthen their resilience to emerging health threats.

## Data Availability

Not applicable as no datasets were generated or analysed for this work.

## Supplementary Data

75000 Supplement
