# Supplementary material for: Timely evidence generation in epidemic response: lessons from an attempted analytical epidemiological investigation during an mpox outbreak, Ireland, 2022
Source: Euro Surveill. 2026 Oct 1;31(39):2600170. doi: 10.2807/1560-7917.ES.2026.31.39.2600170 (PMC13633468; doi:10.2807/1560-7917.ES.2026.31.39.2600170)
Supplement: Supplementary file 1 [file 26-00170_NIELSEN_Supplement.pdf]

Disclaimer: This supplementary material is hosted by *Eurosurveillance* as supporting information alongside the article "Timely evidence generation in epidemic response: lessons from an attempted analytical epidemiological investigation during an mpox outbreak, Ireland, 2022", on behalf of the authors, who remain responsible for the accuracy and appropriateness of the content. The same standards for ethics, copyright, attributions and permissions as for the article apply. Supplements are not edited by *Eurosurveillance* and the journal is not responsible for the maintenance of any links or email addresses provided therein.

Supplementary Table S1. Preparedness and implementation checklists for analytical epidemiological investigations during public health emergencies

**Checklist 1. Collaboration and stakeholder engagement**	
☐	Engage stakeholders at the earliest stage of an investigation including relevant health services, community groups and key populations, and ensure active stakeholder engagement throughout.
☐	Clarify roles and responsibilities from the outset to avoid confusion or delays during critical response phases.
☐	Maintain and invest in long-term relationships with NGOs and community partners between emergencies to preserve trust and facilitate rapid activation during crises, including established contact lists, designated liaison persons, and predefined approaches for involving affected communities in investigation design, communication strategies and recruitment pathways.
☐	Leverage pre-existing partnerships and relationships to enable community-focused and culturally appropriate approaches.
☐	Acknowledge pre-existing work pressures and limitations in human and operational capacity, ensuring realistic expectations between stakeholders to manage resources effectively.
☐	Allocate sufficient and sustained human and financial resources to support meaningful stakeholder involvement and partnership continuity. Examples of preparedness actions may include maintaining updated stakeholder mapping, establishing communication pathways with key community partners, and developing templates for early engagement during emerging outbreaks.
**Checklist 2. Governance and legal clarity**	
☐	Develop and disseminate clear (national) guidance outlining statutory functions and legal frameworks supporting epidemiological investigations during outbreaks.
☐	Establish a shared understanding among stakeholders on the distinction between statutory public health functions and research.
☐	Create and maintain resources including decision trees to distinguish epidemiological investigations from research, template agreements between participating sites, governance approval pathways, and communication materials clarifying the legal remit to facilitate operationalisation of governance frameworks during future investigations.
**Checklist 3. Operational readiness and investigation logistics**	
☐	Maintain readily available technical expertise within outbreak investigation teams to manage survey systems and troubleshoot problems promptly.
☐	Identify potential logistical bottlenecks in advance, including translation and site-agreement processes, and incorporate mitigation plans into preparedness frameworks.
☐	Develop adaptable questionnaire modules and pre-translated standard survey instruments using languages relevant to key populations for outbreak scenarios. These could include demographic, exposure, behavioural, and clinical modules.
☐	Maintain pre-tested digital survey platforms, including validated survey templates, text-based invitations, standard operating procedures (SOPs) for data management, and troubleshooting pathways for technical failures.

☐	Integrate the routine use of investigation tools, platforms, and survey instruments into non-emergency public health activities to maintain system functionality, ensure resources remain up to date, and sustain staff familiarity for rapid activation during outbreak responses.
☐	Establish a repository of investigation resources, including adaptable protocols, questionnaire modules, data dictionaries, analytical code templates and communication materials, to enable rapid adaptation during future emergencies.
**Checklist 4. Preparedness planning and anticipation of evidence needs**	
☐	Integrate simulation exercises (SIMEX) focused on rapid outbreak investigations into routine national preparedness activities. Exercises should test the full investigation pathway, including identification of evidence needs, activation of governance processes, recruitment procedures, data collection platforms, analysis workflows and dissemination of findings.
☐	Establish a dedicated operational unit or mechanism within the public health system to coordinate analytical investigations during emergencies.
☐	Incorporate early discussions of evidence needs into the initial stages of outbreak response planning to ensure timely data collection and informed decision-making.
☐	Maintain readiness to propose and launch investigations even when case numbers are low, to capture early insights and real-time evidence generation.
☐	Align national preparedness activities, where appropriate, with established international investigation frameworks (e.g., standardised study protocols and readiness exercises) to facilitate rapid study activation and cross-country comparability.
**Checklist 5. Human resources and surge capacity planning**	
☐	Establish dedicated surge capacity arrangements at national level, including rosters of trained staff (e.g., Consultants in Public Health Medicine, epidemiologists, data managers, statisticians, communication specialists) who can be rapidly deployed to support analytical investigations. These mechanisms could include cross-institutional agreements with universities, research networks or partner public health agencies, while also leveraging European surge capacity mechanisms, such as the European Union Health Task Force (EUHTF), where appropriate.
☐	Strengthen analytical epidemiology capacity within routine workplans to ensure a pool of experienced personnel is available during emergencies.
☐	Allocate on-site resources within health services to support the implementation of emergency investigations.
☐	Foster collaboration across governmental, academic, and community partners to share expertise and capacity, and alleviate workforce pressures during health crises.
☐	Draw on existing models such as specialist preparedness teams and multi-partner research networks (8, 14) to enhance surge capacity and workforce resilience. Examples of existing surge capacity models include dedicated rapid response teams that combine epidemiologists, laboratory specialists, behavioural scientists and data experts who can be deployed during health emergencies. Adapted models could include national rosters, standing agreements with academic partners, or regional/EU-level support mechanisms.
☐	Develop and maintain readily accessible national and EU-wide outbreak investigation toolkits with standardised protocols, resources and best practices to streamline mobilisation and improve preparedness.
